# Effects of processing method on chemical compositions and nutritional quality of ready‐to‐eat sea cucumber (*Apostichopus japonicus*)

**DOI:** 10.1002/fsn3.921

**Published:** 2019-01-22

**Authors:** Meng Li, Yanxia Qi, Lin Mu, Zhibo Li, Qiancheng Zhao, Jing Sun, Qinghua Jiang

**Affiliations:** ^1^ College of Food Science and Engineering Dalian Ocean University Dalian China; ^2^ Liaoning Provincial Aquatic Products Analyzing Testing and Processing Technology Scientific Service Centre Dalian China; ^3^ Dalian Caishendao group company Dalian China

**Keywords:** nutritional quality, ready‐to‐eat product, sea cucumber, true retention value

## Abstract

The effect of commercial processing methods on the nutritional value of ready‐to‐eat (RTE) sea cucumber *Apostichopus japonicas* was examined in this study. RTE sea cucumber products named RTE‐T and RTE‐V were prepared by two commercial methods, traditional processing, and vacuum cooking, respectively. Proximate, polysaccharide and mineral element composition, amino acid profiles, and true retention values of RTE sea cucumber products were evaluated and compared. Both commercial processing methods significantly changed the nutrient composition in RTE products, except that of Zn and Cu. Comparison of true retention values among RTE products showed that novel commercial method of vacuum cooking resulted in lower nutrient loss and had a shorter processing time than traditional processing. However, soaking after vacuum cooking significantly increased the nutrient loss of RTE sea cucumber. Therefore, vacuum cooking without soaking may be a promising alternative for producing RTE sea cucumber products with high nutritional quality.

## 1 INTRODUCTION

Sea cucumber has long been traditionally consumed as a medicine and tonic food in Asia and the Middle East (Kiew & Don, 2012). Previous studies have been confirmed that therapeutic properties and medicinal benefits of sea cucumber were linked to many bioactive substances such as glycoprotein, glycosphingolipids, and peptides (Hu, Wang, Wang, Xue, & Wang, 2017; Hu, Wang, Wang, Li et al., 2017; Hu et al., 2018; Wang et al., 2016). In China, approximately 28 of the 134 species of sea cucumber are considered edible (Zhang et al., 2016). In order to meet the increasing demand, the sea cucumber culture technology was developed in China in the 1980s and has matured over the past several decades. In recent years, sea cucumber *Apostichpous japonicas* has become the dominate species cultured in northern China, including in Liaoning and Shandong Provinces. *Apostichopus japonicus* production in China exceeded 137 thousand tons in 2011. The farmed *A. japonicus* is rich in proteins, minerals, and special bioactive substances such as polysaccharides (Gao et al., 2016).

Sea cucumbers are usually processed for consumption by a series of procedures, such as boiling, salting, drying, and soaking. Most of sea cucumbers are processed into dried or salted products to facilitate storage and transport. These products have to be rehydrated in cold water for more than 2 days and then subjected to a complex cooking procedure before consuming (Ferdouse, 2004; Ji, Kim, Dong, Pan, & Yoon, 2014). Due to the complicated protocol of cooking, the dried or salted products, commercialized ready‐to‐eat (RTE) sea cucumber products have gained popularity in China in recent years.

Salted sea cucumber with its low cost and simplicity of processing has generally been used starting material for the production of RTE sea cucumber (Yang et al., 2015). Traditionally, fresh sea cucumbers are processed into salted material by boiling and salting and then processed into RTE by rehydration and repeated boiling for 3–5 days. This traditional process for preparing RTE products is a time‐consuming and labor‐intensive task, and more importantly, it leads to the loss of many nutrients (Mujumdar, 2007). Therefore, an economical and quick processing method, suitable for industrial use, is required. Sous‐vide cooking or vacuum cooking seems to be a promising method to meet the requirements of industrial production. Vacuum cooking is defined as cooking raw materials under controlled conditions of temperature and time, inside heat‐stable vacuumed pouches (Schellekens, 1996). This cooking method can improve the shelf‐life and enhance the taste and nutritional value of meat (Baldwin, 2012). If this method is used to process fresh sea cucumbers, the processing cycle for RTE products could be shortened to half a day. In addition, eliminating the repeated boiling and soaking may lower the nutrients loss. However, there is little scientific cooking sea cucumber.

True retention values (TRVs) have been previously used as indicators of the effects of processing on the nutrients retention in processed foods (Badiani et al., 2013; Ersoy & Özeren, 2009; Maurizio, Silvia, Vittoria, Alessandra, & Anna, 2010). TRVs are defined as calculations based on the nutrient content of known weight of food before and after processing (Murphy, Criner, & Gray, 1975). Although the retained nutrient content is a special concern of consumers while choosing sea cucumber products, there are very little data showing the nutrient retention patterns of sea cucumbers processed by different methods. Thus far, there have been reports on the nutrient content of only dried or fresh sea cucumber (Bechtel, Oliveira, Demir, & Smiley, 2013; Li, Li, Guo, Li, & Zhu, 2015; Wen, Hu, & Fan, 2010; Zhang et al., 2016). However, the TRVs of differently processed sea cucumbers are yet to be determined, especially for the RTE products, which have shown a trend of increasing sales volume.

The aim of this work was to investigate the effect of different processing methods, in particular vacuum cooking, on the proximate composition and polysaccharide and the mineral and amino acid profiles of sea cucumber (*A. japonicus*). This research also provides information on the effect of rehydration treatment on the instant products based on vacuum cooking method. TRVs of RTE products were measured to determine the optimal processing method, since high TRVs are linked to the conservation of nutrients in sea cucumber and are desirable in term of RTE sea cucumber economics.

## 2 MATERIALS AND METHODS

### 2.1 Raw material

Farmed Sea cucumbers (*A. japonicus*) were harvested by hands from the Huanghai sea near Changhai county, Dalian, China. Live sea cucumbers were iced and shipped to the local seafood company (Dalian Caishendao Co., Ltd., Dalian, China) for processing within 12 hr after harvesting.

### 2.2 Processing

**Figure 1:**
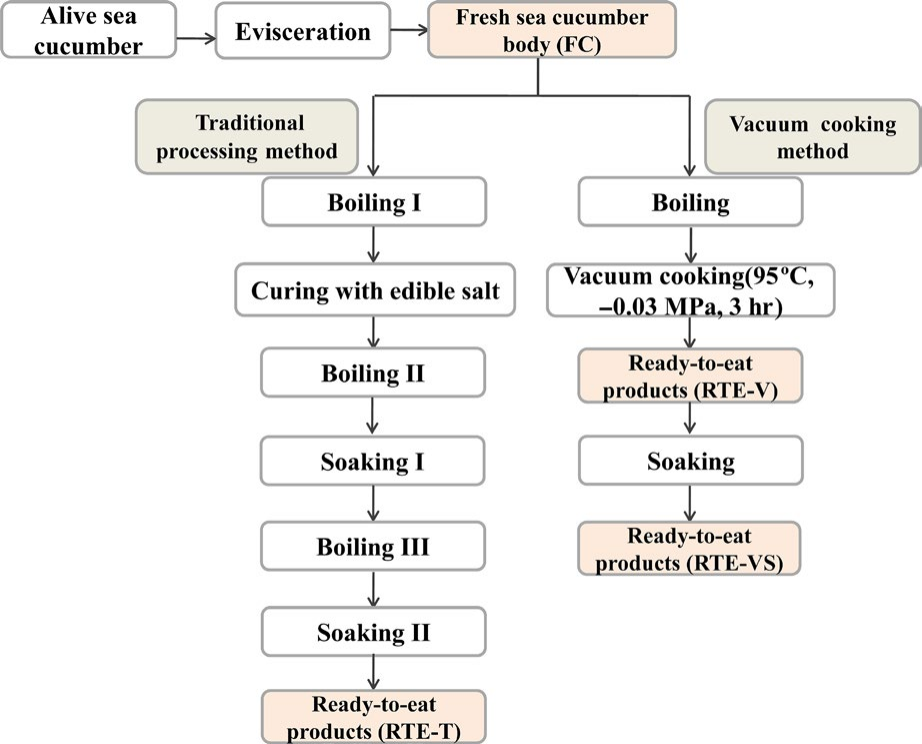
The processing procedures of sea cucumber in this study

Figure 1 shows the methods used for sea cucumber processing in this study. Approximately 80 kg of live sea cucumber was used as the starting material. Approximately 250 g of each of the samples of the live sea cucumbers were eviscerated and washed, thus obtaining approximately 50 kg fresh sea cucumber body wall. This material was divided into two groups. One group, named fresh sea cucumber (FC) with 5 kg sea cucumber body wall, was directly frozen in the freezer. The other group of about 45 kg sea cucumber body wall was used for producing RTE products.

#### 2.2.1 RTE products prepared by traditional processing in the northern part of China (RTE‐T)

Approximately 15 kg fresh sea cucumber body wall was boiled in hot water for about 20 min, followed by curing with edible salt (3:1 w/w) for 12 hr. The salted samples were boiled in fresh water for about 90 min and then soaked in 4°C distilled water for 20 hr to reduce the salt content. These samples were cleaned and boiled in purified water for 20 min, followed by soaking for 40 hr to finally obtain RTE products named RTE‐T.

#### 2.2.2 RTE products prepared by vacuum cooking (RTE‐V)

Approximately 30 kg fresh sea cucumber body wall was boiled in fresh water for about 15 min. The boiled material was subjected to vacuum cooking, using the modified heat‐stable vacuumized machine. The machine consisted of a steam jacketed kettle and a pressure cooker vessel with an inner plate. The sea cucumber body walls were spread in the pressure cooker vessel. The machine settings selected for processing were as follows: heating temperature of jacketed kettle was 95°C, vacuum pressure of vacuum pan was set to −0.04 MPa, and heating time was 3 hr. The samples were removed at the end of vacuum cooking under these conditions and cleaned with purified water to obtain RTE products named RTE‐V.

#### 2.2.3 Rehydrated RTE‐V products

To get bigger volume of RTE sea cucumber, approximately 10 kg of the RTE‐V samples were soaked in 4°C distilled water for 12 hr to obtain rehydrated products named RTE‐VS.

All the RTE samples were frozen, packed, and stored at −20°C. The samples were packed in foam boxes and transported to food laboratory of Dalian Ocean University, Dalian, China.

### 2.3 Proximate composition analysis

Proximate compositions of moisture, protein, fat, and ash of all the samples were measured by the methods of Association of Official Analytical Chemists as described below (AOAC 2005). Moisture content was determined by oven‐drying method (method 952.08); protein was determined by the method of Kjeldahl (method 981.10), and protein content was calculated as 6.25 times % N; fat was determined by petroleum ether extraction using the Soxhlet apparatus by the method 991.36; ash contents were measured by incineration in a muffle furnace at 550°C for 24 hr (method 938.08). Carbohydrate content was determined by the phenol–sulfuric acid method (Dubois, Gilles, Hamilton, Rebers, & Smith, 1955).

### 2.4 Polysaccharide analysis

Polysaccharides were prepared according to the method described by Vieira with slight modification (1988).The powders of sea cucumber samples were immersed in acetone and kept at 4°C for 24 hr. The dried sample was dissolved in 0.1 M sodium acetate buffer (pH 6.0) containing 5% papain, 5 mM EDTA, and 5 mM cysteine and incubated at 60°C for 24 hr. The mixture was centrifuged (2000 × *g* for 10 min at 10°C), and the clear supernatant was collected for subsequent experiments. The reducing sugar content was determined by 3, 5‐dinitro salicylic acid colorimetry (Lindsay, 1973); polysaccharide content is the difference between the contents of total sugars and reducing sugars.

### 2.5 Minerals analysis

Vacuum dried samples were digested in HNO_3_, filtered, and made up to a constant volume as described by Li et al. (2015). The mineral content of the samples was measured using the inductively coupled plasma optical emission spectrometer (ICP‐OES, Optima 8000, Perkin‐Elmer, Waltham, MA, USA). All determinations were performed in triplicate.

### 2.6 Amino acid analysis

Amino acid content was determined using ISO 13903:2005 method as described below (ISO 2005). The samples were hydrolyzed with 6 M HCl in drying oven at 110°C for 24 hr and then diluted with ultrapure water. The diluted samples were evaporated in a rotary evaporator at 40–50°C and dissolved in sodium citrate buffer (pH 2.2). These samples were applied to an L‐8800 amino acid analyzer (HITACHI, Japan). The tryptophan content was measured using colorimetric method, following alkaline hydrolysis (Hugli & Moore, 1972).

The essential amino acid score (EAAS) was calculated according to the FAO/WHO/UNU (2007) reference amino acid requirements of adults:

EAAS = (Amount of essential amino acids in the test protein/Amount of essential amino acids in FAO referred protein) × 100

### 2.7 True retention value (TRV) determinations

The TRVs were calculated according to the method described by Murphy et al. (1975) and using the following formula:

%TRV = [(nutrient content per g of processed sea cucumber × g of sea cucumber after processing)/(nutrient content per g of fresh sea cucumber body wall × g of fresh sea cucumber body wall before processing)] × 100

### 2.8 Statistical analysis of results

SPSS13.0 statistical package (SPSS Inc., Chicago, IL, USA) was used for statistical analysis of the results. Final values are presented as means. Standard error is indicated by error bars. The analysis of variance (ANOVA) and Student's *t* test were used to determine the significance, and the means were considered statistically significant when *p *<* *0.05. OriginPro 8.5 software (OriginLab, Northampton, MA, USA) was used to prepare the figures.

## 3 RESULTS AND DISCUSSION

### 3.1 Proximate composition

The basic compositional traits and content of selected nutrients in raw (FC) and three types of RTE products (RTE‐V, RTE‐VS, and RTE‐T) are shown in Table 1. The contents of the basic components, namely moisture, protein, fat, ash, and carbohydrate in fresh *A. japonicus* from Dalian, China in this study were similar to those reported in previous investigations of *A. japonicus* from Qingdao, China (Dong, Dong, Tian, Wang, & Zhang, 2006).

**Table 1:** Proximate composition, contents of polysaccharides, and selected minerals in FC and RTE sea cucumber products

Unit		FC	RTE‐V	RTE‐VS	RTE‐T
g/100 g, wet weight basis	Moisture	91.71 ± 0.23^c^	80.96 ± 0.32^d^	94.11 ± 0.13^b^	95.55 ± 0.32^a^
	Protein	3.35 ± 0.15^c^	13.30 ± 0.51^a^	4.68 ± 0.10^b^	3.88 ± 0.57^c^
	Fat	0.29 ± 0.04^b^	0.88 ± 0.12^a^	0.25 ± 0.02^b^	0.15 ± 0.02^c^
	Ash	2.97 ± 0.01^a^	2.42 ± 0.01^b^	0.30 ± 0.01^c^	0.27 ± 0.00^d^
	Carbohydrates	1.11 ± 0.10^b^	2.75 ± 0.12^a^	0.83 ± 0.04^c^	0.60 ± 0.06^d^
g/100 g, dry weight basis	Polysaccharide	6.75 ± 0.59^b^	8.25 ± 0.73^a^	7.08 ± 1.74^b^	6.10 ± 1.69^b^
mg/100 g, dry weight basis	Na	18271.34 ± 449.21^a^	12014.65 ± 530.53^b^	4777.07 ± 183.72^c^	4932.76 ± 245.65^c^
	K	1660.15 ± 57.52^a^	340.62 ± 4.86^b^	90.57 ± 1.11^c^	49.96 ± 0.67^d^
	Ca	1506.92 ± 12.22^a^	1354.91 ± 7.61^b^	1283.12 ± 37.04^b^	1115.97 ± 49.65^c^
	Mg	1480.44 ± 36.51^a^	696.37 ± 29.79^b^	473.05 ± 10.39^c^	184.40 ± 1.21^d^
	Fe	26.54 ± 3.34^a^	10.87 ± 2.11^b^	10.23 ± 1.47^b^	14.46 ± 3.84^b^
	Mn	0.65 ± 0.09^a^	0.46 ± 0.08^b^	0.56 ± 0.05^ab^	0.63 ± 0.07^a^
	Cr	0.67 ± 0.07^a^	0.37 ± 0.07^b^	0.24 ± 0.07^b^	0.32 ± 0.04^b^
	Zn	5.02 ± 0.95^a^	4.24 ± 0.66^a^	4.83 ± 0.45^a^	3.87 ± 0.27^a^
	Cu	1.14 ± 0.13^a^	1.05 ± 0.15^a^	1.03 ± 0.11^a^	1.13 ± 0.12^a^

*Notes*

Results are mean ± standard deviation (*n* = 3), different superscript letters in the same row represent statistical differences at *p *<* *0.05.

FC, fresh sea cucumber as control; RTE‐V, ready‐to‐eat products prepared by vacuum cooking; RTE‐VS, ready‐to‐eat products prepared by vacuum cooking and soaking; RTE‐T, ready‐to‐eat products prepared by traditional processing.

Fresh sea cucumber had a higher moisture content and lower protein content, compared to those in other species, including *Cucumaria frondosa* (Zhong, Khan, & Shahidi, 2007), *Holothuria scabra* (Özer, Mol, & Varlık, 2004), *Acaudina molpadioides*, and *Thelenota ananas* (Chen, 2003). Moisture content was decreased by the RTE‐V method of processing and increased by RTE‐VS and RTE‐T methods. RTE‐V methods increased the protein content.

The fat content of *A. japonicus*‐FC was lower than that of *Cucumaria frondosa* (Zhong et al., 2007) and higher than those of *Acaudina molpadioides* and *Thelenota ananas* (Chen, 2003). RTE‐VS method of processing increased, and RTE‐T method decreased the fat content.

The ash content of *A. japonicus*‐FC was similar to that of *Parastichopus californicus* (Chang‐Lee, Price, & Lampila, 1989), *Cucumaria frondosa* (Zhong et al., 2007), and *Thelenota ananas* (Chen, 2003), and higher than that of *Acaudina molpadioides* (Chen, 2003). Within the proximate composition, generalized and significant decrease in all RTE sea cucumber products compared to those of FC products was observed only in ash.

The carbohydrate content of *A. japonicus*‐FC was higher than that in the other reported sea cucumber species, including *Parastichopus parvimensis* (Chang‐Lee et al., 1989)*, Parastichopus californicus* (Chang‐Lee et al., 1989)*,* and *Acaudina molpadioides* (Chen, 2003). Carbohydrate content was increased by RTE‐V and decreased by RTE‐VS and RTE‐T.

RTE‐V, when compared to RTE‐VS or RTE‐T, had a lower moisture content and higher contents of other basic components (*p *<* *0.05). Protein, ash, and carbohydrate contents in RTE‐V were higher than those reported for rehydrated sea cucumber, *Cucumaria frondosa* and canned *Parastichopus parvimensis* (Chang‐Lee et al., 1989; Zhong et al., 2007). RTE‐VS, the rehydrated products of RTE‐V, also had significantly higher contents of basic components than those in RTE‐T, except moisture (*p *<* *0.05).

### 3.2 Polysaccharides contents

Table 1 shows polysaccharide content in FC and RTE products. The polysaccharide content in FC was 6.75 ± 0.59 g/100 g, which was in agreement with the results obtained by Gao et al. (2016). The polysaccharides isolated from *A. japonicus* have potential biological properties such as antifatigue, antithrombotic, anticancerous, and antiosteoarthritic properties (Bordbar, Anwar, & Saari, 2011). RTE‐V samples showed a significant increase in the polysaccharide content (*p *<* *0.05). There was no significant difference in the polysaccharide contents of RTE‐VS and RTE‐T samples (*p *>* *0.05).

### 3.3 Selected mineral contents

The content of four macro elements [sodium(Na); potassium(K); calcium(Ca); magnesium(Mg)] and five trace elements [iron(Fe); manganese(Mn); chromium(Cr); zinc(Zn); copper(Cu)] were determined in FC and RTE products (Table 1). These minerals are necessary for the regulation of essential functions in the human body (Karimiankhosroshahi, Hosseini, Rezaei, Khaksar, & Mahmoudzadeh, 2015).

Table 1 shows the contents of Na, K, Ca, and Mg, in decreasing order, in FC. Na and K contents in FC were higher than those previously reported by Li and Lee et al. for *A. japonicus* (Lee et al., 2014; Li et al., 2015). Ca and Mg contents in FC were in agreement with the results obtained by Lee et al. (2014). The mineral content of sea cucumber was significantly affected by processing, as in the case of cooked fish and meat (Koubaa, Abdelmouleh, Bouain, & Mihoubi, 2014; Oz, Aksu, & Turan, 2016). All the RTE products exhibited significantly decreased levels of the macro mineral nutrients studied (*p *<* *0.05). Our findings were similar to those of Li et al., who reported that the mineral content of sea cucumber (*Apostichopus japonicas selenka*) processed by the drying and rehydration methods decreased significantly (Li et al., 2015).

Among the selected trace elements, Fe was found to be most abundant in FC, followed by Zn. When compared to the values reported by Liu et al. for *A. japonicus* from Zhangzidao island, Dalian, Fe and Mn contents in FC were found to be similar, Zn content was higher, and Cr and Cu contents were lower (Liu et al., 2012). All the RTE products of sea cucumber displayed a significant decrease in Fe and Cr contents, relative to those in FC. Decreased Mn content was observed in RTE‐V only. There was no significant effect of processing on Zn and Cu content (*p *>* *0.05). According to Gokoglu et al. and Karimian‐Khosroshahi et al., boiling or microwave processing of rainbow trout did not significantly change the content of Zn and Cu (Gokoglu, Yerlikaya, & Cengiz, 2004; Karimiankhosroshahi et al., 2015).

Compared to RTE‐VS or RTE‐T, RTE‐V had significantly higher macro mineral nutrient content with the exception of Ca(*p *<* *0.05), and similar trace mineral content, with the exception of Mn. RTE‐VS, when compared to RTE‐T, had significantly higher contents of K, Ca, and Mg. There was no difference between RTE‐T and RTE‐VS in the contents of the other minerals studied.

### 3.4 Amino acid composition

The amino acid (AA) content of FC and RTE products are presented in Table 2. All the RTE sea cucumber products had significantly higher content of AAs compared to that of FC, with the exception of cysteine (*p *<* *0.05). The most abundant AA in FC was glutamic acid, followed by glycine, which is the case with most identified sea cucumber species (Wen et al., 2010). The concentration of glycine was the highest followed by glutamic acid, in RTE products. According to Bordbar et al. (2011), glycine and glutamic acid play a significant role in immune regulation. Glutamic acid content in FC, RTE‐V, RTE‐VS, and RTE‐T was 5.53 ± 0.15, 8.08 ± 0.22, 8.76 ± 0.02, and 10.35 ± 0.02 g/100 g, respectively, which was similar to that observed in raw and cooked fish (Erkan, Özden, & SelçUk, 2010; Zhao, Zhuang, Song, Shi, & Zhang, 2010). Glycine contents in FC, RTE‐V, RTE‐VS, and RTE‐T was 4.86 ± 0.07, 8.86 ± 0.30, 10.85 ± 0.21, and 11.65 ± 0.21 g/100 g, respectively, which was much higher than that in raw fish, *P. punctatissimus* (Zhao et al., 2010), raw and steamed *Anchovy*,* Small bluefish*, and *Medium bluefish* (Erkan et al., 2010).

**Table 2:** Amino acid profiles (g/100g, dry weight basis) of FC and RTE sea cucumber products

	FC	RTE‐V	RTE‐VS	RTE‐T
Asp	4.05 ± 0.09^d^	5.89 ± 0.18^c^	6.89 ± 0.11^b^	7.54 ± 0.10^a^
Thr*	2.11 ± 0.03^c^	2.84 ± 0.10^b^	3.50 ± 0.11^a^	3.67 ± 0.02^a^
Ser	1.88 ± 0.06^d^	2.79 ± 0.12^c^	3.14 ± 0.03^b^	3.57 ± 0.09^a^
Glu	5.53 ± 0.15^c^	8.08 ± 0.22^b^	8.76 ± 0.02^a^	10.35 ± 0.02^a^
Pro	2.81 ± 0.08^d^	5.26 ± 0.05^c^	5.87 ± 0.21^b^	6.96 ± 0.06^a^
Gly	4.86 ± 0.07^d^	8.86 ± 0.30^c^	10.85 ± 0.21^b^	11.65 ± 0.21^a^
Ala	2.28 ± 0.06^d^	3.70 ± 0.11^c^	4.41 ± 0.01^a^	4.88 ± 0.02^a^
Val*	1.73 ± 0.01^d^	2.33 ± 0.06^c^	2.64 ± 0.02^b^	2.93 ± 0.05^a^
Met*	0.78 ± 0.05^c^	1.16 ± 0.01^b^	1.21 ± 0.04^b^	1.59 ± 0.01^a^
Ile*	1.51 ± 0.03^c^	2.05 ± 0.03^b^	2.06 ± 0.03^b^	2.60 ± 0.06^a^
Leu*	2.13 ± 0.01^d^	2.78 ± 0.08^c^	2.96 ± 0.04^b^	3.48 ± 0.07^a^
Tyr	1.05 ± 0.08^c^	1.45 ± 0.14^b^	1.61 ± 0.03^b^	1.78 ± 0.05^a^
Phe*	1.34 ± 0.01^c^	1.76 ± 0.06^b^	1.88 ± 0.11^b^	2.22 ± 0.06^a^
Lys*	1.85 ± 0.04^c^	1.97 ± 0.05^b^	2.36 ± 0.11^a^	2.33 ± 0.04^a^
His	0.53 ± 0.01^d^	0.61 ± 0.02^c^	0.75 ± 0.02^a^	0.73 ± 0.01^a^
Arg	2.87 ± 0.05^d^	4.43 ± 0.17^c^	5.33 ± 0.15^b^	5.8 ± 0.08^a^
Cys	0.61 ± 0.04^b^	0.61 ± 0.01^b^	0.88 ± 0.21^a^	0.67 ± 0.03^a^
Trp*	0.38 ± 0.01^d^	0.40 ± 0.01^c^	0.54 ± 0.01^a^	0.48 ± 0.00^b^
Lys/Arg	0.64 ± 0.01^a^	0.44 ± 0.01^b^	0.44 ± 0.01^b^	0.40 ± 0.01^c^

*Notes*

Results are mean ± standard deviation (*n* = 3), the values in the same raw with different superscript letter are significantly different (*p *<* *0.05).

FC, fresh sea cucumber as control; RTE‐V, ready‐to‐eat products prepared by vacuum cooking; RTE‐VS, ready‐to‐eat products prepared by vacuum cooking and soaking; RTE‐T, ready‐to‐eat products prepared by traditional processing.

* Essential amino acids.

In addition to the abundance of glutamic acid and glycine, another important feature of amino acid composition in sea cucumber is the low lysine/arginine ratio. (Bordbar et al., 2011). The rations of lysine to arginine in FC, RTE‐V, RTE‐VS, and RTE‐T are about 0.64, 0.44, 0.44, and 0.40, respectively. Our results concur with those of Wen et al. (2010), who reported that the lysine/arginine ration of sea cucumber was lower than that in other sea food products such as fish, crab, and shrimp. It has been well documented that proteins with low lysine/arginine ratio have hypocholesterolemic effects (Sugano, Ishiwaki, & Nakashima, 1984).

Threonine, valine, methionine, isoleucine, leucine, phenylalanine, lysine, and tryptophan are generally regarded as essential amino acids for humans (Belitz, Grosch, & Schieberle, 2009). According to Table 2, essential amino acid (EAA) contents of RTE products were significantly higher (*p *<* *0.05) than those in FC. The essential amino acid score (EAAS) is a key factor in the evaluation of the nutritional quality of food products. EAAS in FC and RTE products shown in Table 3 were determined as per the FAO/WHO/UNU standards. The essential amino acids, which are most often limiting, are lysine, methionine, threonine, and tryptophan (Bingham, 1978). The only limiting essential amino acid in FC was leucine. Wen et al. suggested that lysine and methionine are the limiting essential amino acids in eight dried sea cucumber products (Wen et al., 2010). Leucine, valine, and lysine were the limiting amino acids in all RTE products.

**Table 3:** Essential amino acid scores of FC and RTE sea cucumber products

Essential amino acid	FC	RTE‐V	RTE‐VS	RTE‐T	Reference^a^
	Score	Score	Score	Score	Content (mg/g protein)
Thr	226	178	191	204	23
Val	110	85	85	95	39
Met+Cys	155	118	118	132	22
Ile	123	97	87	110	30
Leu	90	68	63	76	59
Phe+Tyr	197	153	147	170	30
Lys	102	62	67	67	45
Trp	150	100	117	100	6

*Notes*

Abbreviations are as in Table 2.

a Reference amino acid pattern of adults.

### 3.5 Nutrient retention in the RTE sea cucumbers

**Figure 2:**
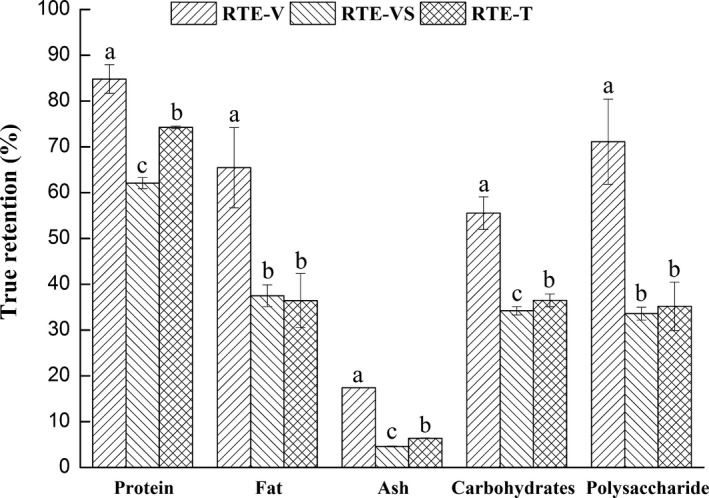
True retention values of proximate composition and polysaccharide of RTE sea cucumber products. ▨, ready‐to‐eat products prepared by vacuum cooking (RTE‐V); ▧, ready‐to‐eat products prepared by vacuum cooking and soaking (RTE‐VS); ▩, ready‐to‐eat products prepared by traditional processing (RTE‐T); Error bars show standard error. Values within each set of columns are significantly different (*p *<* *0.05) if they do not have a common letter

**Figure 3:**
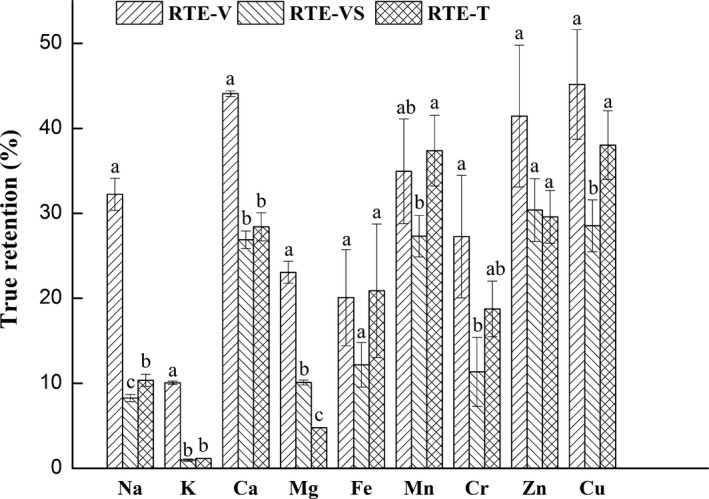
True retention values of mineral content of RTE sea cucumber products. ▨, ready‐to‐eat products prepared by vacuum cooking (RTE‐V); ▧, ready‐to‐eat products prepared by vacuum cooking and soaking (RTE‐VS); ▩, ready‐to‐eat products prepared by traditional processing (RTE‐T); Error bars show standard error. Values within each set of columns are significantly different (*p *<* *0.05) if they do not have a common letter

In order to examine how the selected nutrients were affected by processing, their TRVs were calculated and shown in Figures 2 and 3. As shown in Figure 2, the ranges of the basic nutrients retained in RTE‐V, RTE‐VS, and RTE‐T were 17% to 84%, 4% to 64%, and 6% to 72%, respectively. The results showed that processing greatly influenced the proximate composition of sea cucumber. The reason could be explained by previous studies, which reported that proteins (Lang, 1970), fats (Lang, 1970), ash (Kumar & Aalbersberg, 2006), and carbohydrates (Chen et al., 2015) in food were lost into the drip or wastewater during processing. TRVs of protein, fat, carbohydrate, and ash in RTE sea cucumbers in descending order were protein > fat > carbohydrate > ash. Thus, the retention of ash was the lowest in RTE products (Figure 2). This result was in agreement with that reported by Fukunaga, Matsumoto, Murakami, and Hatae (2004) who showed that the ash of dried sea cucumber was eluted rapidly into the soaking water. RTE‐V had the highest basic nutrient retention among the three RTE products. However, TRVs of protein and ash in RTE‐VS were lower (*p *<* *0.05) than those in RTE‐T, which demonstrated that soaking after vacuum cooking resulted in further loss of protein and ash.

As shown in Figure 2, TRVs of polysaccharides were the highest in RTE‐V, at approximately 71.1%. TRV of polysaccharides dropped dramatically to 33.6% in RTE‐VS. This result demonstrated that soaking after vacuum cooking aggravated the loss. Loss of polysaccharides during processing could be explained by their high solubility in water. Chen et al. reported that active components like crude polysaccharides were detected largely in the waste fluids generated while processing the sea cucumber (Chen et al., 2015). There was no difference (*p *>* *0.05) in the TRVs of polysaccharides between RTE‐VS and RTE‐T.

Among the minerals studied, TRVs in three RTE products were less than 50% (Figure 3), which demonstrated that minerals were lost more easily during processing. Badiani et al. (2013) reported that the TRVs of minerals in European sea bass decreased significantly upon cooking. RTE‐V products displayed higher (*p *<* *0.05) TRVs for macro minerals (Na, K, Ca, and Mg) than those of RTE‐VS and RTE‐T. The differences in TRVs of trace minerals (Fe, Mn, Cr, Zn, and Cu) between RTE‐V and RTE‐T were insignificant (*p* ˃ 0.05). However, RTE‐VS had higher TRV for Mg and lower TRVs for Na, Mn, and Cu compared to those of RTE‐T, indicating that soaking after vacuum cooking resulted in further loss of Na, Mn, and Cu.

For amino acids, TRVs ranged from 49% to 91%, 40% to 70%, and 40% to 69% for RTE‐V, RTE‐VS, and RTE‐T, respectively (Table 4). RTE‐V had significantly higher (*p *<* *0.05) AA TRVs compared to those of RTE‐VS and RTE‐T. More proline and glycine and less cysteine and lysine were retained in all the RTE products. TRVs of all the EAAs were lower than those for non‐EAAs, with the exception of cysteine and histidine, in the three RTE products. TRVs of all EAAs, with the exception of methionine, were below 55% in both RTE‐VS and RTE‐T. These results indicate that EAAs were more likely to be lost, compared to non‐EAAs during processing.

**Table 4:** True retention values (%) of amino acids of RTE sea cucumber products

Amino acid	RTE‐V	RTE‐VS	RTE‐T
Asp	71.40 ± 2.15^a^	53.79 ± 0.88^b^	54.97 ± 0.20^b^
Thr*	66.05 ± 2.30^a^	52.31 ± 1.59^b^	51.18 ± 0.19^b^
Ser	72.70 ± 3.14^a^	52.74 ± 0.48^c^	55.59 ± 0.65^b^
Glu	71.73 ± 1.94^a^	50.04 ± 0.12^c^	54.76 ± 0.37^b^
Pro	91.77 ± 0.86^a^	65.91 ± 2.30^c^	69.85 ± 0.00^b^
Gly	89.47 ± 3.00^a^	70.50 ± 1.38^b^	68.80 ± 0.42^c^
Ala	79.71 ± 2.29^a^	61.14 ± 0.01^c^	64.14 ± 0.00^b^
Val*	66.10 ± 1.61^a^	48.10 ± 0.39^c^	51.63 ± 0.12^b^
Met*	73.46 ± 0.90^a^	49.10 ± 1.44^c^	61.82 ± 0.26^b^
Ile*	66.63 ± 0.92^a^	43.08 ± 0.59^c^	52.15 ± 0.27^b^
Leu*	64.05 ± 1.96^a^	43.88 ± 0.63^c^	50.09 ± 0.10^b^
Tyr	67.77 ± 6.61^a^	48.42 ± 0.85^c^	51.01 ± 0.78^b^
Phe*	64.70 ± 2.08^a^	44.35 ± 2.51^c^	52.59 ± 1.69^b^
Lys*	52.27 ± 1.32^a^	40.31 ± 1.82^b^	40.41 ± 0.11^b^
His	56.02 ± 1.96^a^	44.39 ± 1.26^b^	43.70 ± 0.00^b^
Arg	75.88 ± 2.91^a^	58.69 ± 1.64^b^	58.61 ± 0.29^b^
Cys	49.08 ± 0.57^a^	45.67 ± 1.11^b^	44.02 ± 2.03^b^
Trp*	52.35 ± 0.00^a^	45.47 ± 0.00^b^	44.78 ± 0.00^c^

*Notes*

Results are mean ± standard deviation (*n* = 3), the values in the same raw with different superscript letter are significantly different (*p *<* *0.05).

Abbreviations are as in Table 2. *Essential amino acids.

## 4 CONCLUSION

Comparison of raw sea cucumber with sea cucumber processed by different methods showed that the basic nutrients, polysaccharides, amino acids, and the minerals examined in this study, with the exception of Zn and Cu, differed significantly by the processing method. TRVs of the various nutrients indicated that ready‐to‐eat processing methods resulted in varying degrees of nutrient loss. Among the processed sea cucumber products, vacuum cooking not only produced RTE‐V with the highest content of protein, fat, ash, polysaccharide, minerals (Na, K, Ca, and Mg), and amino acids but also yielded the highest TRVs for all the chemical and biochemical components studied, with the exception of trace minerals (Fe, Mn, Cr, Zn, and Cu). However, soaking after vacuum cooking significantly increased the nutrient loss. TRVs of protein, ash, and minerals (Na, Mn, and Cu) in RTE‐VS were lower than those in RTE‐T. Therefore, we concluded that the vacuum cooking methods, in general, could be considered better than the traditional processing methods in preserving the nutritional quality of sea cucumber.

## CONFLICT OF INTEREST

On behalf of all authors, the corresponding author states that there is no conflict of interest.

## ETHICAL STATEMENT

This article does not contain any studies with human participants or animals requiring ethical approval.
